# Supplementary material for: Nucleus-translocated GCLM promotes chemoresistance in colorectal cancer through a moonlighting function
Source: Nat Commun. 2025 Jan 2;16:263. doi: 10.1038/s41467-024-55568-1 (PMC11696352; doi:10.1038/s41467-024-55568-1)
Supplement: Supplementary file 1 — Supplementary information [file 41467_2024_55568_MOESM1_ESM.pdf]

Supplementary information

Nucleus-Translocated GCLM Promotes Chemoresistance in Colorectal Cancer through a Moonlighting Function

Jin-Fei Lin, Ze-Xian Liu, Dong-Liang Chen, Ren-Ze Huang, Fen Cao, Kai Yu, Ting Li, Hai-Yu Mo, Hui Sheng, Zhi-Bing Liang, Kun Liao, Yi Han, Shan-Shan Li, Zhao-Lei Zeng, Song Gao, Huai-Qiang Ju, Rui-Hua Xu

Supplementary Figure 1: GCLM depletion enhances the chemosensitivity of CRC cells to oxaliplatin.

Supplementary Figure 2: GCLM translocates to the nucleus up platinum drug treatment.

Supplementary Figure 3: Nuclear GCLM interacts with NKRF to orchestrate NF-κB activity and chemoresistance.

Supplementary Figure 4: Platinum drugs promote GCLM nuclear localization via GCLM binding to importin a5.

Supplementary Figure 5: P38 MPAK-mediated phosphorylation of GCLM participates in platinum drug-induced nuclear localization of GCLM.

Supplementary Figure 6: Phosphorylation of GCLM at T17 contributes to CRC chemoresistance in vivo.

Supplementary Figure 7: Nuclear GCLM is highly expressed in tumours and indicates a poor prognosis.

Supplementary Figure 8: The gating strategy used for apoptosis assays in this study.

Supplementary Table 1: Correlation analysis for clinicopathologic variables in nuclear GCLM expression among 406 CRC patients.

Supplementary Table 2: Univariate and multivariate analysis of prognostic factors for overall survival among 406 CRC patients.

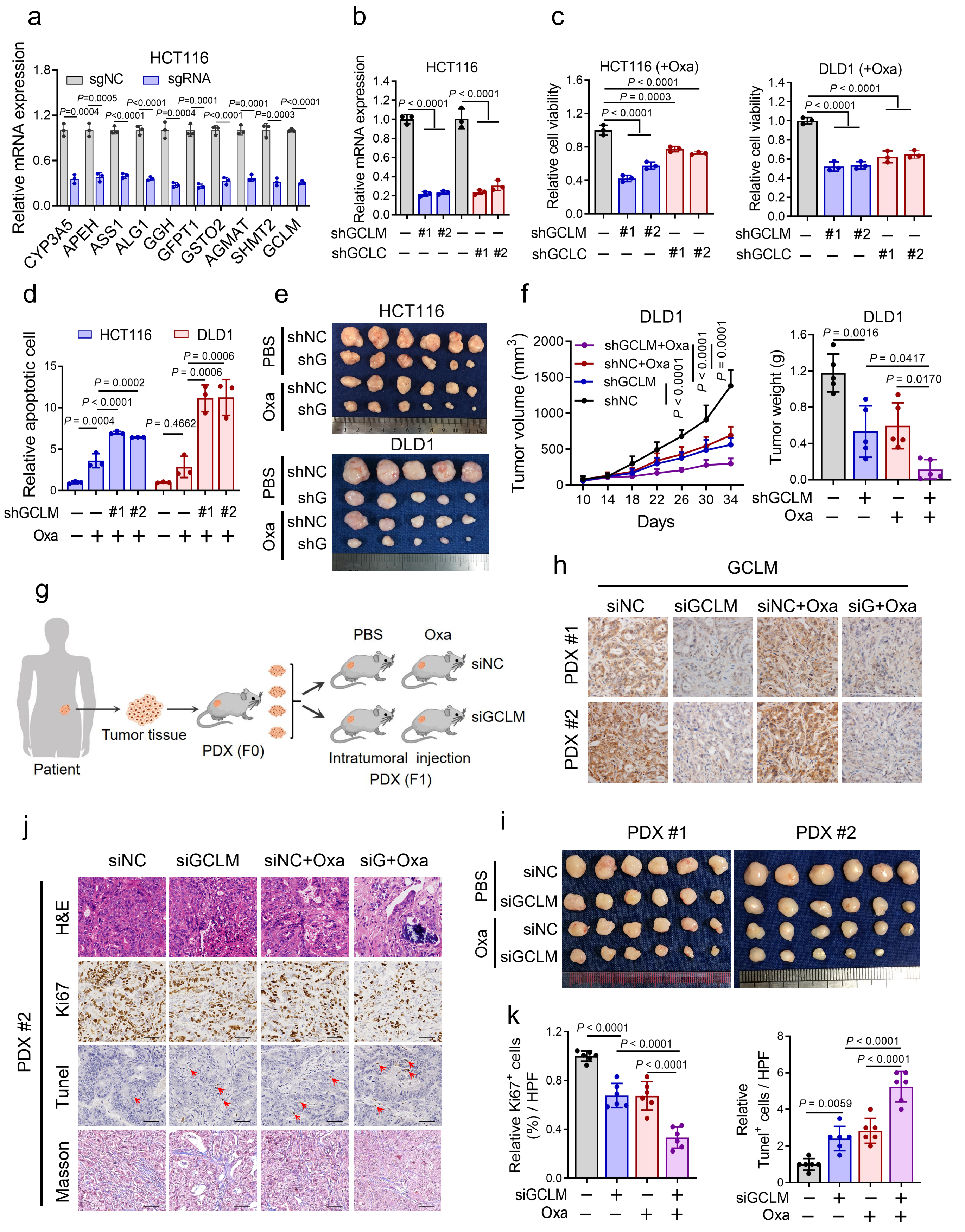
a
HCT116
sgNC
sgRNA
Relative mRNA expression
1.8
1.2
0.6
0.0
P=0.0004
P=0.0005
P<0.0001
P<0.0001
P=0.0004
P=0.0001
P<0.0001
P=0.0001
P=0.0003
P<0.0001
CYP3A5
APEH
ASS1
ALG1
GGH
GFPT1
GSTO2
AGMAT
SHMT2
GCLM
b
HCT116
Relative mRNA expression
P < 0.0001
P < 0.0001
shGCLM
shGCLC
#1
#2
c
HCT116 (+Oxa)
Relative cell viability
P < 0.0001
P = 0.0003
P < 0.0001
DLD1 (+Oxa)
P < 0.0001
P < 0.0001
d
HCT116
DLD1
Relative apoptotic cell
P = 0.0004
P < 0.0001
P = 0.0002
P = 0.4662
P = 0.0006
P = 0.0006
shGCLM
Oxa
e
HCT116
DLD1
PBS
Oxa
shNC
shG
f
DLD1
shGCLM+Oxa
shNC+Oxa
shGCLM
shNC
Tumor volume (mm3)
Days
P < 0.0001
P < 0.0001
P = 0.0001
Tumor weight (g)
P = 0.0016
P = 0.0417
P = 0.0170
g
Patient
Tumor tissue
PDX (F0)
PBS
Oxa
siNC
siGCLM
Intratumoral injection
PDX (F1)
h
GCLM
siNC
siGCLM
siNC+Oxa
siG+Oxa
PDX #1
PDX #2
i
PDX #1
PDX #2
PBS
Oxa
siNC
siGCLM
j
siNC
siGCLM
siNC+Oxa
siG+Oxa
PDX #2
H&E
Ki67
Tunel
Masson
k
Relative Ki67+ cells (%) / HPF
P < 0.0001
P < 0.0001
P < 0.0001
Relative Tunel+ cells / HPF
P = 0.0059
P < 0.0001
P < 0.0001
siGCLM
Oxa

Supplementary Fig. 1 GCLM depletion enhances the chemosensitivity of CRC cells to oxaliplatin.

a Q-PCR analysis of the candidate gene expression after knocking out CYP3A5, APEH, ASS1, ALG1, GGH, GFPT1, GSTO2, AGMAT, SHMT2 or GCLM in HCT116 cells. **b** Q-PCR analysis of GCLM or GCLC expression after knocking down GCLM or GCLC in HCT116 cells. **c** Viability of HCT116 or DLD1 cells after knocking down GCLM or GCLC with oxaliplatin treatment (Oxa, 40 μM for HCT116, 80 μM for DLD1, 24 h). **d** Quantification analysis of the percentages of apoptotic cells in **Figure 1 e**. Photographs of the excised tumors from HCT116 and DLD1 cells based CDX models (HCT116, n = 6 per group; DLD1, n = 5 per group). **f** Statistical analysis of CDX tumor volume and weight in nude mice after implantation of GCLM-knockdown or control DLD1 cells ($2x10^6$), followed by intraperitoneal injections of PBS or oxaliplatin (5 mg/kg). **g** Illustration of the methodology used to establish CRC PDX models. **h** IHC staining showing GCLM in PDX-based paraffin-embedded subcutaneous tumor sections. Scale bar = 50 μm. **i** Photographs of the excised tumors from PDX #1 (left) and PDX #2 (right) models (n =6 per group). **j** H&E, IHC staining of Ki67, Tunel assay and Masson's trichrome staining in PDX #2-based paraffin-embedded subcutaneous tumor sections. The red arrowheads indicate the positive cell of Tunel staining. Scale bar =50 μm. **k** Quantification of the proliferation index (Ki67 staining) and apoptotic index (Tunel assay) of the PDX #2 models. n = 3 biologically independent experiments in **a-d,** n = 5 mice in **f** and n = 6 mice in **k**. All the data are presented as the mean ± S.D. The *P* values were calculated by two-tailed unpaired Student's t test (**a**), one-way ANOVA (**b, c, d, f** right and **k**), and two-way ANOVA (**f** left).

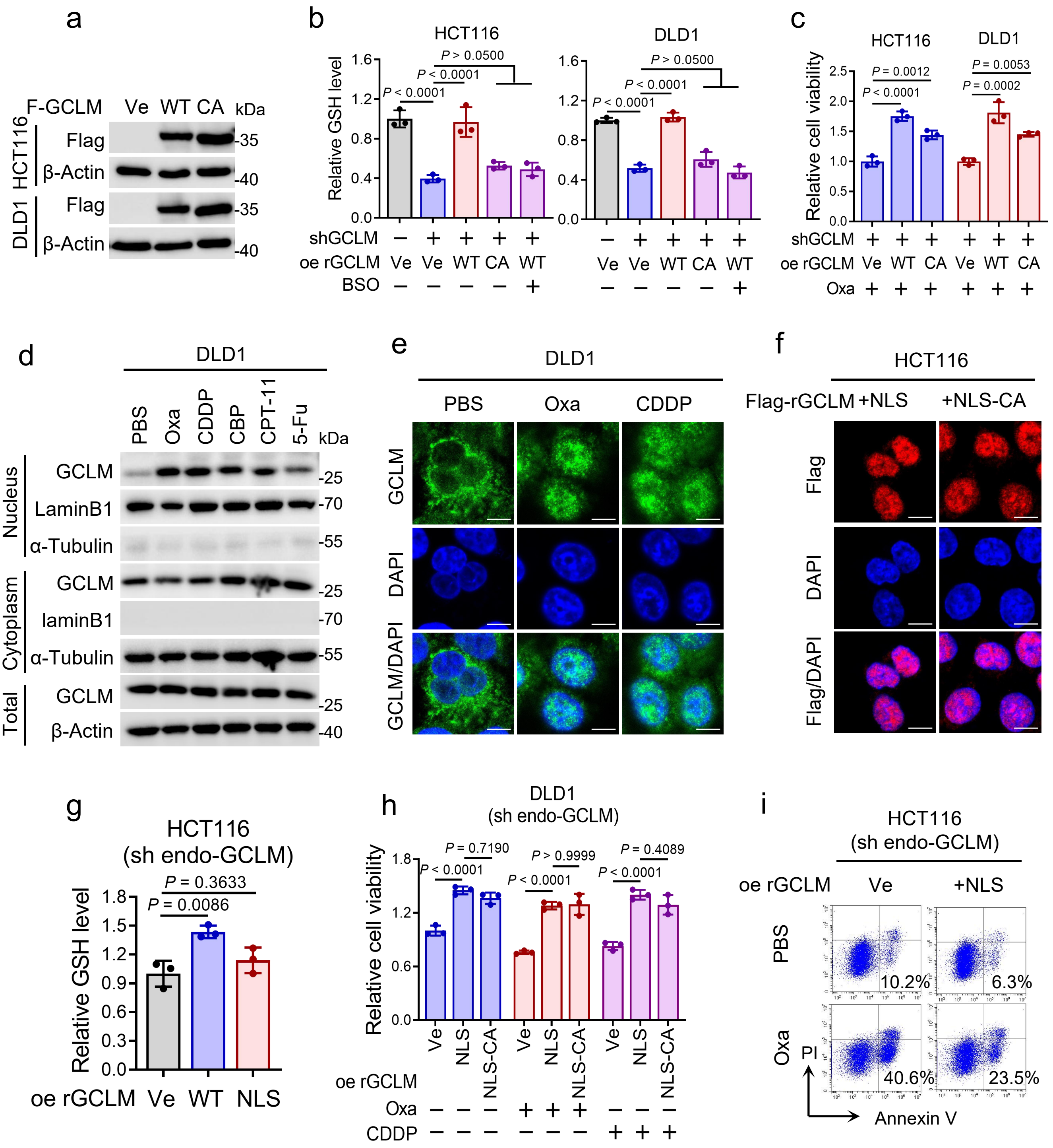
a
F-GCLM Ve WT CA kDa
HCT116
Flag
β-Actin
DLD1
Flag
β-Actin
-35
-40
b
HCT116
DLD1
Relative GSH level
P > 0.0500
P < 0.0001
shGCLM
oe rGCLM Ve Ve WT CA WT
BSO
c
HCT116
DLD1
Relative cell viability
P = 0.0012
P = 0.0053
P = 0.0002
shGCLM
oe rGCLM Ve WT CA Ve WT CA
Oxa
d
DLD1
PBS Oxa CDDP CBP CPT-11 5-Fu kDa
Nucleus
GCLM
LaminB1
α-Tubulin
Cytoplasm
GCLM
laminB1
α-Tubulin
Total
GCLM
β-Actin
e
DLD1
PBS Oxa CDDP
GCLM
DAPI
GCLM/DAPI
f
HCT116
Flag-rGCLM +NLS +NLS-CA
Flag
DAPI
Flag/DAPI
g
HCT116
(sh endo-GCLM)
Relative GSH level
P = 0.3633
P = 0.0086
oe rGCLM Ve WT NLS
h
DLD1
(sh endo-GCLM)
Relative cell viability
P = 0.7190
P > 0.9999
P = 0.4089
P < 0.0001
Ve NLS NLS-CA
oe rGCLM
Oxa
CDDP
i
HCT116
(sh endo-GCLM)
oe rGCLM Ve +NLS
PBS
Oxa
10.2%
6.3%
40.6%
23.5%
PI
Annexin V

Supplementary Fig. 2 GCLM translocates to the nucleus up platinum drug treatment.

a IB analysis of GCLM protein expression in HCT116 and DLD1 cells overexpressing control, rGCLM WT or C193/194A mutant (CA). **b** The GSH level analysis of endogenous GCLM-knockdown HCT116 and DLD1 cells overexpressing control, rGCLM WT or C193/194A mutant (CA) with or without the treatment of BSO (150 μM, 24 h). **c** Cell viability of endogenous GCLM-knockdown HCT116 and DLD1 cells overexpressing control, rGCLM WT or C193/194A mutant in the presence of oxaliplatin (40 μM for HCT116 and 80 μM for DLD1, 24 h). **d** IB detection of total, cytoplasmic and nuclear GCLM expression in DLD1 cells treated with chemotherapeutic drugs including oxaliplatin (Oxa, 80 μM), cisplatin (CDDP, 80 μM), carboplatin (CBP, 80 μM), irinotecan (CPT-11, 40 μM) and 5-fluorouracil (5-FU, 40 μM) for 24 h. **e** IF staining showing the localization of GCLM in DLD1 cells with the treatment of oxaliplatin or cisplatin (80 μM, 24 h). Scale bar = 10 μm. **f** IF staining images of the localization of GCLM in HCT116 cells overexpressing nuclear GCLM (+NLS) or C193/194A mutant (+NLS-CA). Scale bar =10 μm. **g** The GSH level analysis of GCLM knockdown HCT116 cells overexpressing control, rGCLM WT or nuclear GCLM (rGCLM NLS). **h** Cell viability of GCLM-knockdown DLD1 cells overexpressing control, nuclear GCLM WT (rGCLM NLS) or C193/194A mutant (CA) with PBS, oxaliplatin or cisplatin treatment (80 μM, 24 h). **i** Annexin V/PI analysis the apoptotic cells in GCLM-knockdown HCT116 cells overexpressing control, nuclear GCLM (rGCLM NLS) with or without oxaliplatin treatment (40 μM, 24 h). IB experiments were repeated three times and n = 3 biologically independent experiments in **b, c, g, h.** All the data are presented as the mean ± S.D. The *P* values were calculated by one-way ANOVA (**b, c, g**) and two-way ANOVA (**h**).

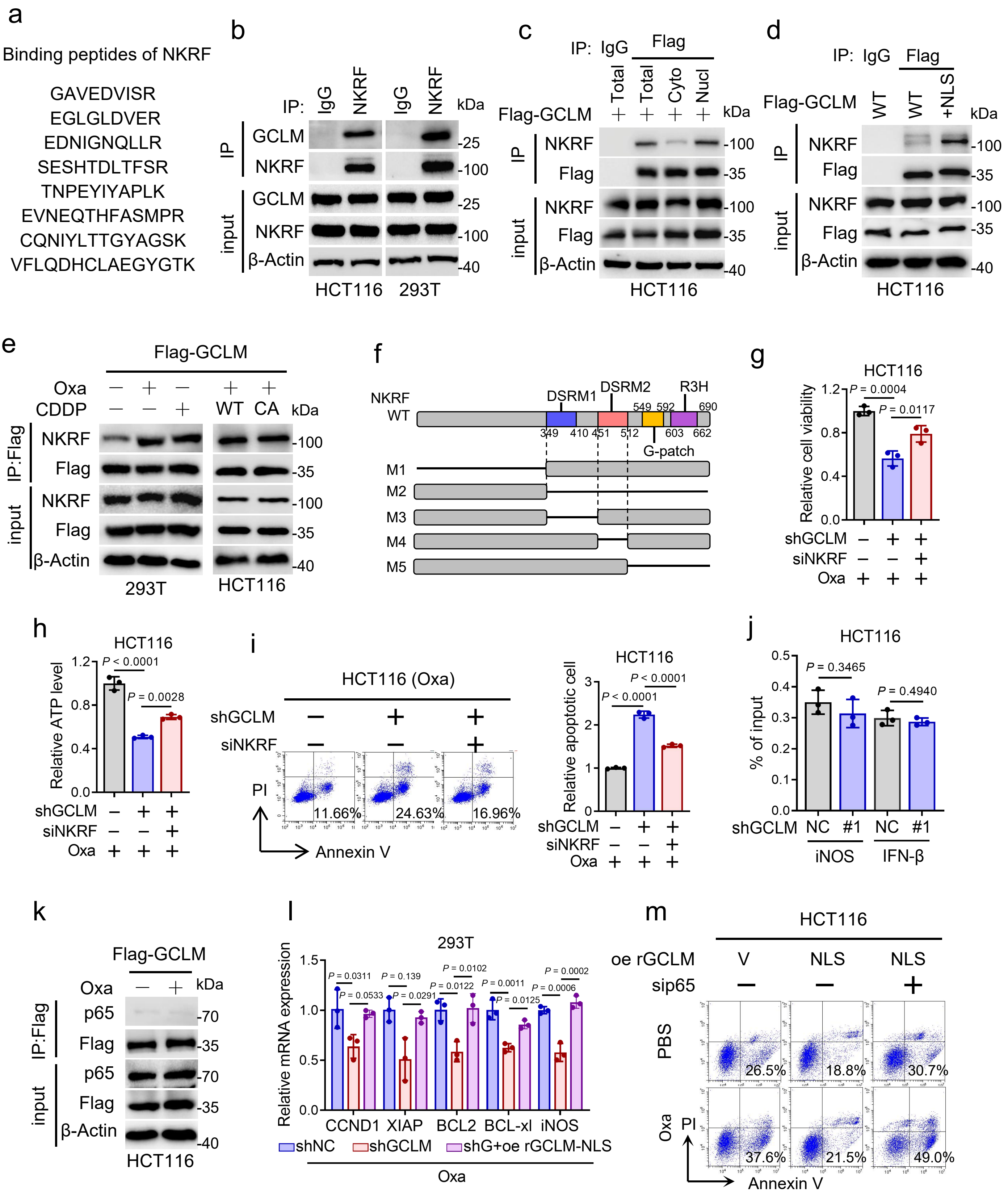
a
Binding peptides of NKRF
GAVEDVISR
EGLGLDVER
EDNIGNQLLR
SESHTDLTFSR
TNPEYIYAPLK
EVNEQTHFASMPR
CQNIYLTTGYAGSK
VFLQDHCLAEGYGTK
b
IP: IgG NKRF IgG NKRF kDa
IP GCLM NKRF
input GCLM NKRF β-Actin
-25 -100 -25 -100 -40
HCT116 293T
c
IP: IgG Flag
Total Total Cyto Nucl
Flag-GCLM + + + + kDa
IP NKRF Flag
input NKRF Flag β-Actin
-100 -35 -100 -35 -40
HCT116
d
IP: IgG Flag
Flag-GCLM WT WT +NLS kDa
IP NKRF Flag
input NKRF Flag β-Actin
-100 -35 -100 -35 -40
HCT116
e
Flag-GCLM
Oxa − + − + +
CDDP − − + WT CA kDa
IP:Flag NKRF Flag
input NKRF Flag β-Actin
-100 -35 -100 -35 -40
293T HCT116
f
NKRF WT
DSRM1 DSRM2 R3H
349 410 451 512 549 592 603 662 690
G-patch
M1 M2 M3 M4 M5
g
HCT116
Relative cell viability
P = 0.0004
P = 0.0117
shGCLM − + +
siNKRF − − +
Oxa + + +
h
HCT116
Relative ATP level
P < 0.0001
P = 0.0028
shGCLM − + +
siNKRF − − +
Oxa + + +
i
HCT116 (Oxa)
shGCLM − + +
siNKRF − − +
PI
Annexin V
11.66% 24.63% 16.96%
HCT116
Relative apoptotic cell
P < 0.0001
P < 0.0001
shGCLM − + +
siNKRF − − +
Oxa + + +
j
HCT116
% of input
P = 0.3465
P = 0.4940
shGCLM NC #1 NC #1
iNOS IFN-β
k
Flag-GCLM
Oxa − + kDa
IP:Flag p65 Flag
input p65 Flag β-Actin
-70 -35 -70 -35 -40
HCT116
l
293T
Relative mRNA expression
P = 0.0311 P = 0.0533 P = 0.139 P = 0.0291 P = 0.0102 P = 0.0122 P = 0.0011 P = 0.0125 P = 0.0002 P = 0.0006
CCND1 XIAP BCL2 BCL-xl iNOS
shNC shGCLM shG+oe rGCLM-NLS
Oxa
m
HCT116
oe rGCLM V NLS NLS
sip65 − − +
PBS
26.5% 18.8% 30.7%
Oxa
37.6% 21.5% 49.0%
PI
Annexin V

Supplementary Fig. 3 Nuclear GCLM interacts with NKRF to orchestrate NF-κB activity and chemoresistance.

a The peptide sequences of NKRF bound by nuclear GCLM in 293T cells under oxaliplatin treatment (40 μM, 24 h) through LC-MS/MS analysis in **Figure 3a**. **b** Co-IP analysis showing the level of GCLM bound by NKRF using anti-NKRF or IgG antibody. **c** Co-IP analysis demonstrating the level of NKRF bound by total, cytoplasmic and nuclear GCLM. **d** Co-IP analysis showing the interaction of GCLM and NKRF in HCT116 cells overexpressing Flag-tagged GCLM WT or nuclear GCLM (+NLS). **e** Co-IP analysis showing the interaction of GCLM and NKRF in 293T cells treated with oxaliplatin (Oxa) or cisplatin (CDDP) (40 μM, 24 h) (left), and in HCT116 cells overexpressing Flag-tagged GCLM WT or C193/194A mutant treated with oxaliplatin (40 μM, 24 h) (right). **f** Schematic illustration of NKRF protein containing four main domains. The strategy for mutating NKRF is also shown. **g-i** GCLM was depleted in HCT116 cells with or without importin α5 inhibition under oxaliplatin treatment (40 μM, 24 h). cell viability (**g**), ATP level (**h**) and apoptotic cells (**i**) were detected. **j** Q-PCR analysis after ChIP analysis showing the occupancy of NKRF on the negative regulatory elements (NRE) of *iNOS* and *IFN-β* promoter. **k** Co-IP analysis showing the interaction of GCLM and p65 in HCT116 cells with or without oxaliplatin treatment (40 μM, 24 h). **l** Q-PCR analysis of the expression of NF-κB/p65-targeted genes (CCND1, XIAP, BCL2, BCL-xl, and iNOS) in 293T cells when depleting GCLM and re-expressiong nuclear GCLM (+NLS) in the presence of oxaliplatin (40 μM, 24 h). **m** Annexin V/PI analysis the apoptotic cells in nuclear GCLM-overexpressed HCT116 cells with control or p65 silencing with or without oxaliplatin treatment (40 μM, 24 h). IB experiments were repeated three times and n = 3 biologically independent experiments in **g-j, l.** All the data are presented as the mean ± S.D. The *P* values were calculated by one-way ANOVA (**g-i, l**) and two-tailed unpaired Student's t test (**j**).

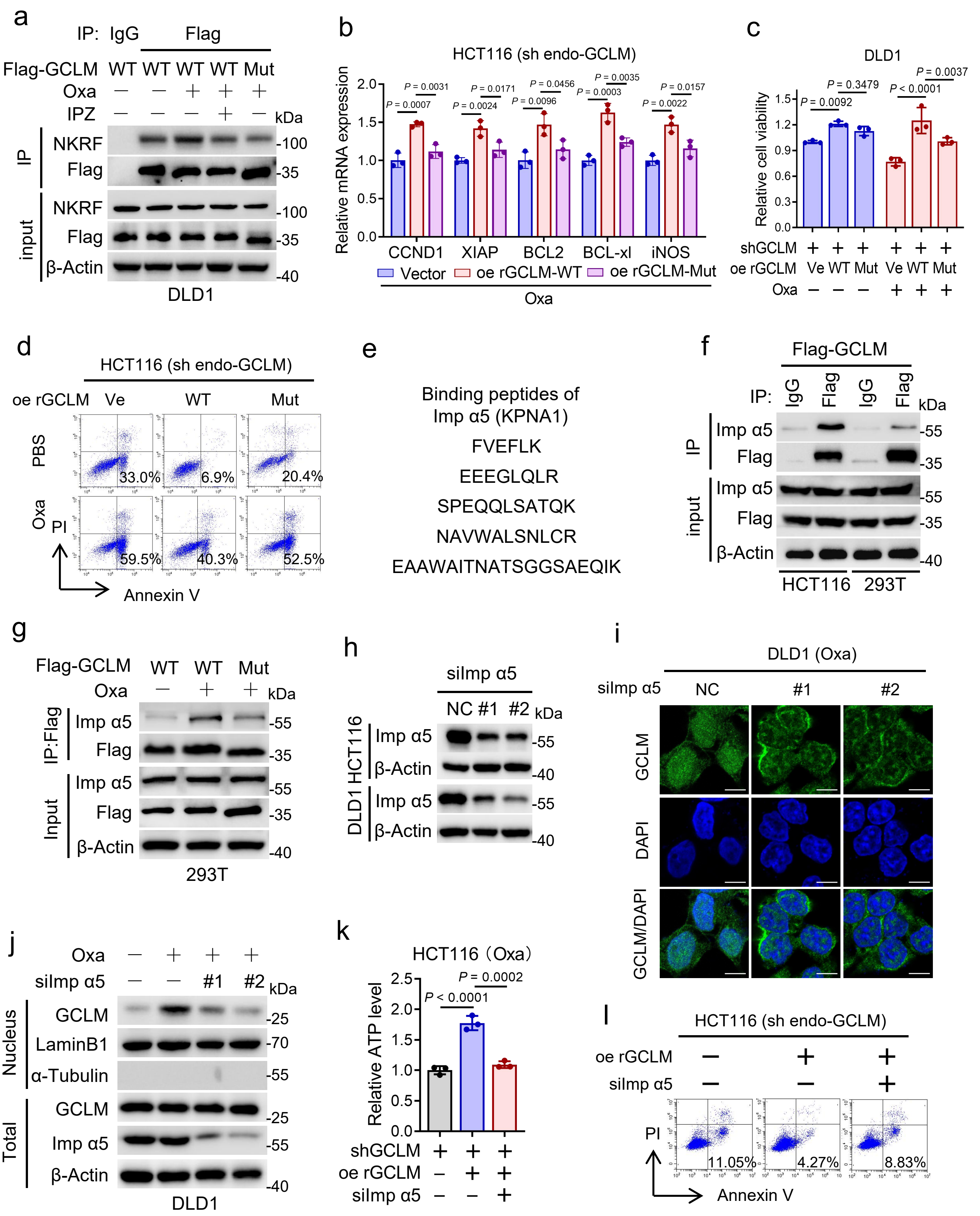

a
IP: IgG Flag
Flag-GCLM WT WT WT WT Mut
Oxa — — + + +
IPZ — — — + —
kDa
IP NKRF -100
Flag -35
input NKRF -100
Flag -35
β-Actin -40
DLD1
b
HCT116 (sh endo-GCLM)
Relative mRNA expression
2.0
1.5
1.0
0.5
0.0
P = 0.0031
P = 0.0007
P = 0.0171
P = 0.0024
P = 0.0456
P = 0.0096
P = 0.0035
P = 0.0003
P = 0.0157
P = 0.0022
CCND1 XIAP BCL2 BCL-xl iNOS
Vector oe rGCLM-WT oe rGCLM-Mut
Oxa
c
DLD1
Relative cell viability
1.5
1.2
0.9
0.6
0.3
0.0
P = 0.0092
P = 0.3479
P < 0.0001
P = 0.0037
shGCLM + + + + + +
oe rGCLM Ve WT Mut Ve WT Mut
Oxa — — — + + +
d
HCT116 (sh endo-GCLM)
oe rGCLM Ve WT Mut
PBS 33.0% 6.9% 20.4%
Oxa 59.5% 40.3% 52.5%
PI
Annexin V
e
Binding peptides of
Imp α5 (KPNA1)
FVEFLK
EEEGLQLR
SPEQQLSATQK
NAVWALSNLCR
EAAWAITNATSGGSAEQIK
f
Flag-GCLM
IP: IgG Flag IgG Flag
kDa
IP Imp α5 -55
Flag -35
input Imp α5 -55
Flag -35
β-Actin -40
HCT116 293T
g
Flag-GCLM WT WT Mut
Oxa — + +
kDa
IP:Flag Imp α5 -55
Flag -35
Input Imp α5 -55
Flag -35
β-Actin -40
293T
h
siImp α5
NC #1 #2
kDa
HCT116 Imp α5 -55
β-Actin -40
DLD1 Imp α5 -55
β-Actin -40
i
DLD1 (Oxa)
siImp α5 NC #1 #2
GCLM
DAPI
GCLM/DAPI
j
Oxa — + + +
siImp α5 — — #1 #2
kDa
Nucleus GCLM -25
LaminB1 -70
α-Tubulin -55
Total GCLM -25
Imp α5 -55
β-Actin -40
DLD1
k
HCT116（Oxa）
Relative ATP level
2.5
2.0
1.5
1.0
0.5
0.0
P = 0.0002
P < 0.0001
shGCLM + + +
oe rGCLM – + +
siImp α5 – – +
l
HCT116 (sh endo-GCLM)
oe rGCLM — + +
siImp α5 — — +
PI
11.05% 4.27% 8.83%
Annexin V

Supplementary Fig. 4 Platinum drugs promote GCLM nuclear localization via GCLM binding to importin a5.

a DLD1 cells overexpressed Flag-tagged GCLM WT or GCLM-NLS Mut with oxaliplatin (80 μM, 24 h) or IPZ treatment (40 μM, 24 h). Co-IP analysis showing the interaction of GCLM and NKRF. **b, c, d** GCLM knockdown HCT116 or DLD1 cells overexpressed vector Flag-tagged GCLM WT or GCLM-NLS Mut with or without oxaliplatin treatment (40 μM, 24 h). Q-PCR analysis of the expression of NF-κB/p65-targeted genes (CCND1, XIAP, BCL2, BCL-xl, and iNOS) (**b**), cell viability (**c**) and apoptotic cells analysis were performed. **e** The peptide sequences of importin α5 bound by total GCLM in HCT116 cells through the LC-MS/MS analysis under oxaliplatin treatment (40 μM, 24 h). **f** HCT116 or 293T cells overexpressed Flag-tagged GCLM WT under oxaliplatin treatment (40 μM, 24 h). Co-IP analysis showing the interaction of importin α5 and GCLM using anti-Flag or IgG antibody. **g** Co-IP analysis showing the GCLM-importin α5 interaction in 239T cells overexpressing Flag-tagged GCLM WT or GCLM-NLS Mut with or without oxaliplatin treatment (40 μM, 24 h). **h** IB analysis the level of importin α5 in control or importin α5 silenced HCT116 and DLD1 cells. **i, j** IF staining showed the localization of GCLM (**i**) and IB detectd nuclear and total GCLM expression (**j**) in DLD1 cells with control or importin α5 silencing in presence or absence of oxaliplatin treatment (80 μM, 24 h). Scale bar =10 μm. **k, l** GCLM knockdown HCT116 cells overexpressed vector or rGCLM WT with control or importin α5 silencing under oxaliplatin treatment (40 μM, 24 h). ATP level (**k**) and apoptotic cells (**l**) were detected. IB experiments were repeated three times and n = 3 biologically independent experiments in **b, c, k**. All the data are presented as the mean ± S.D. The *P* values were calculated by one-way ANOVA (**b**, **k**) and two-way ANOVA (**c**).

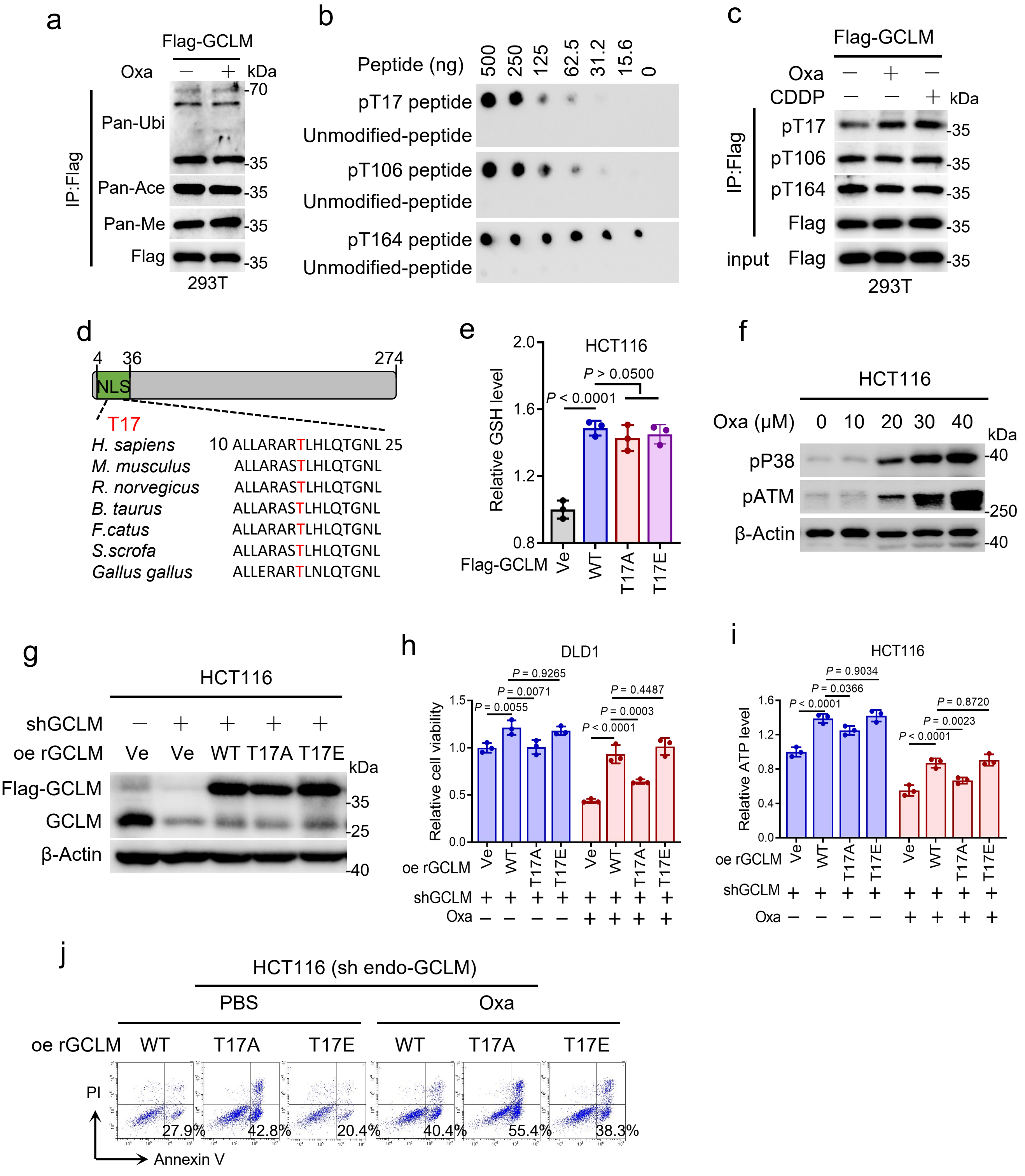

a
Flag-GCLM
Oxa
kDa
IP:Flag
Pan-Ubi
Pan-Ace
Pan-Me
Flag
293T
b
Peptide (ng)
500 250 125 62.5 31.2 15.6 0
pT17 peptide
Unmodified-peptide
pT106 peptide
Unmodified-peptide
pT164 peptide
Unmodified-peptide
c
Flag-GCLM
Oxa
CDDP
kDa
IP:Flag
pT17
pT106
pT164
Flag
input Flag
293T
d
4 36 274
NLS
T17
H. sapiens 10 ALLARARTLHLQTGNL 25
M. musculus ALLARASTLHLQTGNL
R. norvegicus ALLARASTLHLQTGNL
B. taurus ALLARASTLHLQTGNL
F.catus ALLARARTLHLQTGNL
S.scrofa ALLARASTLHLQTGNL
Gallus gallus ALLERARTLNLQTGNL
e
HCT116
Relative GSH level
P > 0.0500
P < 0.0001
Flag-GCLM Ve WT T17A T17E
f
HCT116
Oxa (μM) 0 10 20 30 40
kDa
pP38
pATM
β-Actin
g
HCT116
shGCLM
oe rGCLM Ve Ve WT T17A T17E
kDa
Flag-GCLM
GCLM
β-Actin
h
DLD1
Relative cell viability
P = 0.9265
P = 0.0071
P = 0.0055
P = 0.4487
P = 0.0003
P < 0.0001
oe rGCLM Ve WT T17A T17E Ve WT T17A T17E
shGCLM
Oxa
i
HCT116
Relative ATP level
P = 0.9034
P = 0.0366
P < 0.0001
P = 0.8720
P = 0.0023
P < 0.0001
oe rGCLM Ve WT T17A T17E Ve WT T17A T17E
shGCLM
Oxa
j
HCT116 (sh endo-GCLM)
PBS
Oxa
oe rGCLM WT T17A T17E WT T17A T17E
PI
27.9% 42.8% 20.4% 40.4% 55.4% 38.3%
Annexin V

Supplementary Fig. 5 P38 MPAK-mediated phosphorylation of GCLM participates in platinum drug-induced nuclear localization of GCLM.

a IP analysis demonstrating the common post-translational modifications level of GCLM in 293T cells overexpressing Flag-tagged GCLM and treated with oxaliplatin (40 μM, 24 h), including ubiquitination (Pan-Ubi), acetylation (Pan-Ace) and methylation (Pan-Me). **b** Dot blot analysis showing the specificity of anti-pThr17/Thr106/Thr164 antibodies treated with different concentrations of specific and unmodified peptides. **c** IP analysis showing the level of GCLM phosphorylated at Thr17, Thr106 and Thr164 in 293T cells overexpressing Flag-tagged GCLM and treated with or without oxaliplatin or cisplatin (40 μM, 24 h). **d** Sequence alignment of phosphorylated peptides of GCLM at Thr17 among indicated species. **e** The GSH level analysis of HCT116 cells overexpressing Flag-tagged GCLM WT or T17A, T17E mutants. **f** IB detection of phosphorylated P38 MAPK (pP38) and ATM (pATM) level in HCT116 cells treated with different concentrations of oxaliplatin (24 h). **g** IB detection of GCLM level in control or GCLM knockdown HCT116 cells overexpressing rGCLM WT or T17A, T17E mutants. **h** Cell viability of GCLM knockdown DLD1 cells overexpressing rGCLM WT or T17A, T17E mutants with or without oxaliplatin treatment (80 μM, 24 h). **i, j** ATP level (**i**) and apoptotic cells (**j**) analysis in GCLM knockdown HCT116 cells overexpressing rGCLM WT or T17A, T17E mutants with or without oxaliplatin treatment (40 μM, 24 h). IB experiments were repeated three times and n = 3 biologically independent experiments in **e, h, i**. All the data are presented as the mean ± S.D. The *P* values were calculated by one-way ANOVA (**e**) and two-way ANOVA (**h, i**).

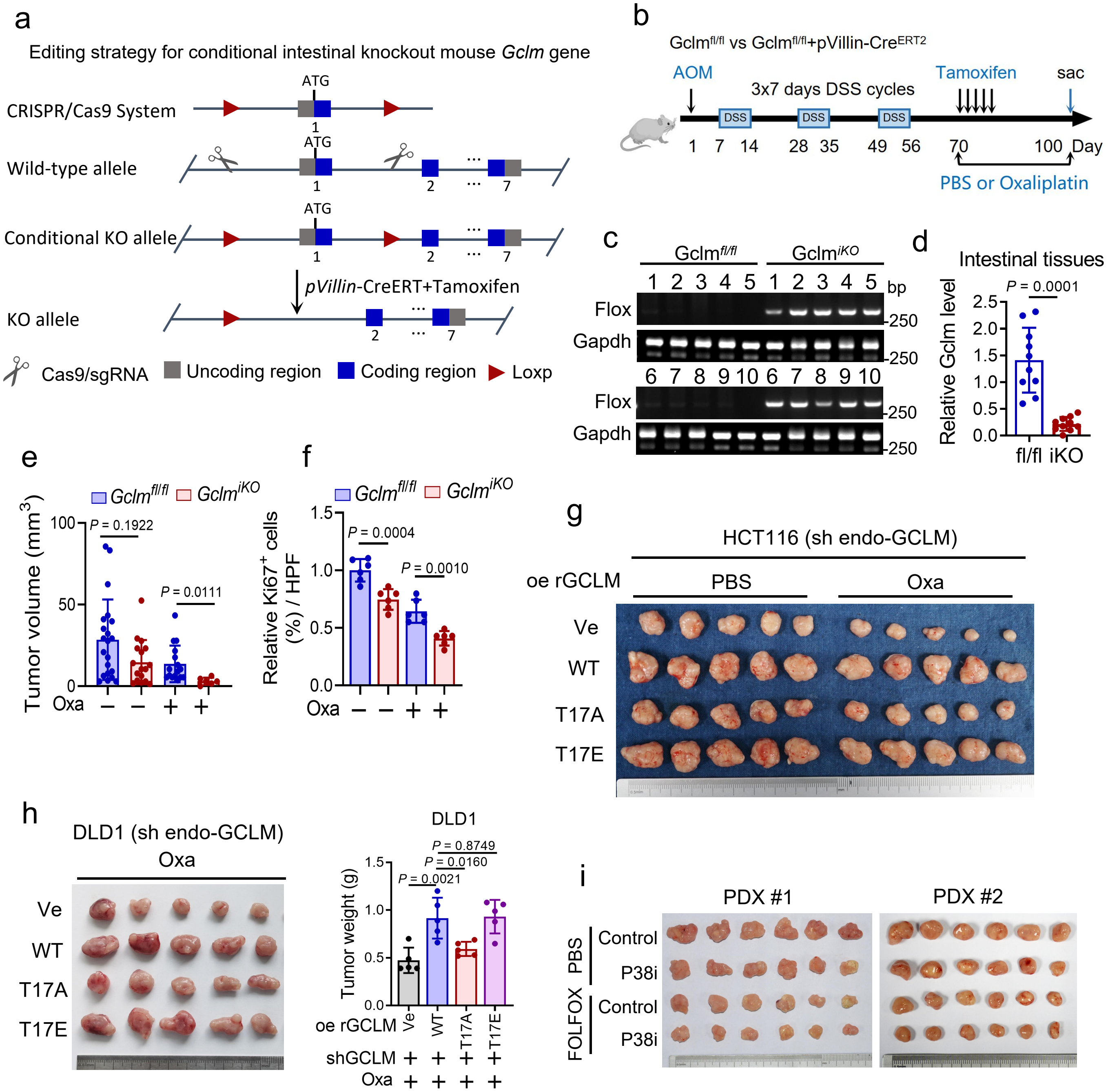

a
Editing strategy for conditional intestinal knockout mouse Gclm gene
CRISPR/Cas9 System
ATG
Wild-type allele
Conditional KO allele
pVillin-CreERT+Tamoxifen
KO allele
Cas9/sgRNA
Uncoding region
Coding region
Loxp
b
Gclm fl/fl vs Gclm fl/fl+pVillin-CreERT2
AOM
3x7 days DSS cycles
Tamoxifen
sac
DSS
1 7 14 28 35 49 56 70 100 Day
PBS or Oxaliplatin
c
Gclm fl/fl
Gclm iKO
bp
Flox
Gapdh
250
d
Intestinal tissues
P = 0.0001
Relative Gclm level
fl/fl iKO
e
Gclm fl/fl Gclm iKO
Tumor volume (mm3)
P = 0.1922
P = 0.0111
Oxa – – + +
f
Relative Ki67+ cells (%) / HPF
P = 0.0004
P = 0.0010
Oxa – – + +
g
HCT116 (sh endo-GCLM)
oe rGCLM
PBS
Oxa
Ve
WT
T17A
T17E
h
DLD1 (sh endo-GCLM)
Oxa
Ve
WT
T17A
T17E
DLD1
Tumor weight (g)
P = 0.8749
P = 0.0160
P = 0.0021
oe rGCLM Ve WT T17A T17E
shGCLM + + + +
Oxa + + + +
i
PDX #1
PDX #2
PBS
Control
P38i
FOLFOX
Control
P38i

Supplementary Fig. 6 Phosphorylation of GCLM at T17 contributes to CRC chemoresistance in vivo.

a Illustration of the editing strategy for conditional intestinal knockout mouse Gclm gene by expressing Cre recombinase under the control of the pVillin gene promoter (pVillin-CreERT2), an intestine-specific promoter. **b** Experimental design for the mouse model of AOM/DSS-induced spontaneous CRC established in $Gclm^{fl/fl}$ and $Gclm^{iKO}$ mice, followed by intraperitoneal injection of PBS or oxaliplatin (5 mg/kg)**.** **c** Agarose gel electrophoresis analysis after PCR analysis of the DNA from mouse intestine tissue, which revealed a 377 bp band after the loxp sequence were cut in the KO allele not the wild-type or floxed allele whose products (7000bp) cannot be amplified. Gapdh was included as a loading control. **d** Q-PCR analysis showing the expression of Gclm in intestine of spontaneous tumors from $Gclm^{fl/fl}$ and $Gclm^{iKO}$ mice after tamoxifen (15 mg/kg for 5 days) treatment. **e, f** Statistical analysis of tumor size of each spontaneous tumor (**e**) and quantification for Ki67 staining (**f**) in $Gclm^{fl/fl}$ and $Gclm^{iKO}$ mice treated with PBS or oxaliplatin (5 mg/kg). **g**, **h** Photographs and weight of CDX tumor after the implantation of endogenous GCLM-knockdown HCT116 (**g**) and DLD1 (**h**) cells ($2x10^6$), which overexpressed rGCLM WT or T17A, T17E mutants, followed by intraperitoneal injections of PBS or oxaliplatin (5 mg/kg) (n = 5 per group). **i** Photographs of PDX models followed by intraperitoneal injections of control or P38 inhibitor (P38i, SB203580, 5 mg/kg) and FOLFOX (oxaliplatin 5 mg/kg, 5-fluorouracil 25 mg/kg) (n = 6 per group). n = 10 mice in **d**, n = 6 mice in **e**, **f** and n = 5 mice in **h.** All the data are presented as mean ± S.D. The *P* values were calculated by two-tailed unpaired Student’s t test (**d**) and one-way ANOVA (**e, f, h** right).

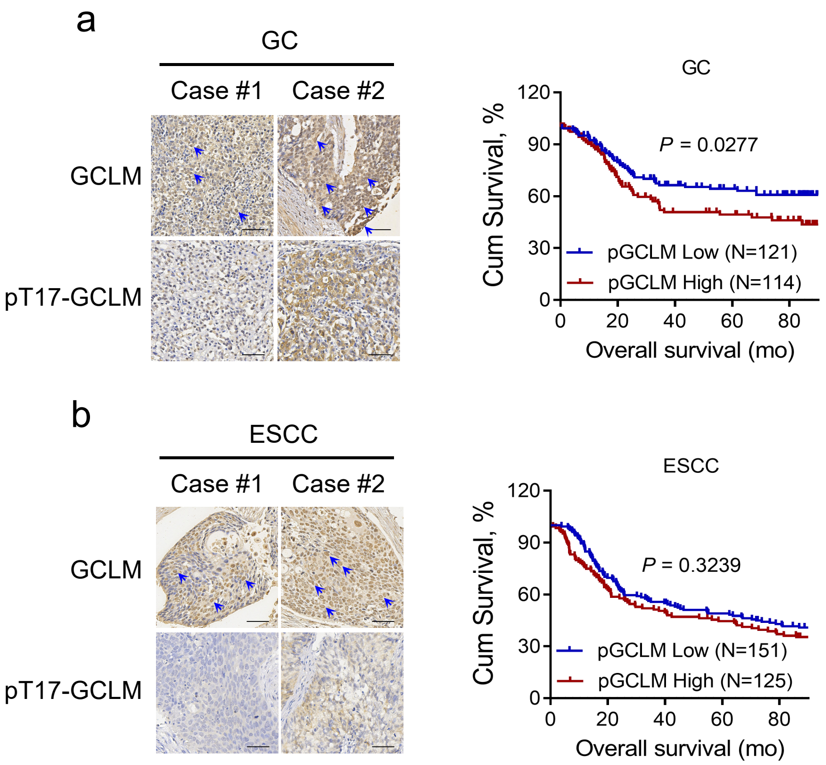

Supplementary Fig. 7 Nuclear GCLM is highly expressed in tumours and indicates a poor prognosis.

a, b IHC staining for nuclear GCLM expressionin (nGCLM, only focusing on GCLM staining in the nucleus, as shown by blue arrowheads) and the level of GCLM phosphorylated at Thr17 (pT17-GCLM) in GC (n = 235, GC tissue specimens) or ESCC (n = 276, ESCC tissue specimens) tissues. GC, gastric carcinoma, ESCC, esophageal squamous cell carcinoma. Scale bar = 50 μm (**a**, **b** left). Overall survival assays of patients with GC or ESCC based on pT17-GCLM expression. We categorized proteins level as low or high compared with the median value of IHC score (**a**, **b** right). The *P* values were calculated by Kaplan-Meier analysis (log-rank test) (**a**, **b** right).

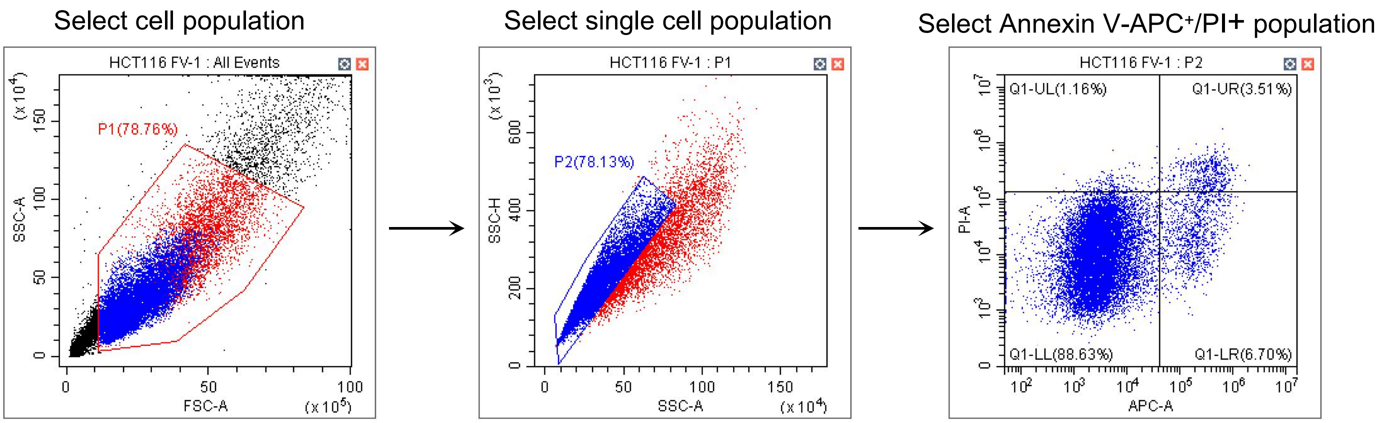

Supplementary Fig. 8 The gating strategy used for apoptosis assays in this study. Apoptosis was analyzed with a Annexin V/PI Kit and conducted with a flow cytometer using APC (638nm) and PI (488nm) channel.

Supplementary Table 1: Correlation analysis for clinicopathologic variables in nuclear GCLM expression among 406 CRC patients

Variable	Low nGCLM n (%)	High nGCLM n (%)	*P* value
Total	183 (45.1)	223 (54.9)	
Age, years			0.690
≤ 57	99 (54.1)	116 (52.0)	
> 57	84 (45.9)	107(48.0)	
Sex			1.000
Male	110 (60.1)	133 (59.6)	
Female	73 (39.9)	90 (40.4)	
Clinical stage			<0.001
Ⅰ-Ⅱ	81 (44.3)	49 (22.0)	
Ⅲ-Ⅳ	102 (55.7)	174 (78.0)	
Histological grade			0.230
Well/moderate	148 (80.9)	169 (75.8)	
Poor and others	35 (19.1)	54 (24.2)	
Tumor depth			0.166
m/sm/mp	21 (11.5)	16 (7.2)	
ss/se/si	162 (88.5)	207 (92.8)	
Vascular invasion			0.416
Absent	127(88.2)	148(84.6)	
Present	17 (11.8)	27 (15.4)	
Perineural invasion			0.647
Absent	84 (58.3)	107 (61.1)	
Present	60(41.7)	68 (38.9)	

The *P* values were determined by two-sided Chi-square test. Abbreviations: m: tumor invasion of mucosa; sm: submucosa; mp: muscular is propria; ss:subserosa; se: serosa penetration; si: invasion to adjacent structures.

Supplementary Table 2: Univariate and multivariate analysis of prognostic factors for overall survival among 406 CRC patients

Factors	Univariate		Multivariate*	
	HR (95% CI)	*P* value	HR (95% CI)	*P* value
Age (<=57/>57)	1.153 (0.750-1.772)	0.516	/	/
Sex (male/female)	1.863(1.177-2.947)	0.008	/	/
TNM stage (Ⅰ-Ⅱ/Ⅲ-Ⅳ)	3.480 (1.998-6.063)	<0.001	/	/
Histological grade (Well,moderate/ Poor and others)	1.743(1.062-2.861)	0.028	1.972 (1.046-3.716)	0.036
Tumor depth (m, sm, mp/ss, se, si)	1.820 (0.776-4.269)	0.168	/	/
Vascular invasion (absent/present)	2.088 (1.015-4.293)	0.045	/	/
Perineural invasion (absent/present)	2.204 (1.243-3.908)	0.007	1.870 (1.021-3.472)	0.043
nGCLM expression (low/high)	1.701 (1.093-2.648)	0.019	2.334 (1.241-4.389)	0.009

The *P* value was determined by the univariate and multivariate cox regression analysis. All the statistical tests were two-sided. Abbreviations: CRC: Colorectal cancer; m: tumor invasion of mucosa; sm: submocosa; mp: muscular is propria; ss:subserose; se: serosa penetration; si: invasion to adjacent structures. HR: hazard ratio; CI: confidence interval.

* For the multivariate model, HR and *P* values were shown by backward method only.
