# Supplementary material for: Nucleus-translocated GCLM promotes chemoresistance in colorectal cancer through a moonlighting function
Source: Nat Commun. 2025 Jan 2;16:263. doi: 10.1038/s41467-024-55568-1 (PMC11696352; doi:10.1038/s41467-024-55568-1)
Supplement: Supplementary file 6 — Reporting Summary [file 41467_2024_55568_MOESM6_ESM.pdf]

nature portfolio

Corresponding author(s): Rui-Hua Xu

Last updated by author(s): Dec 7, 2024

Reporting Summary

Nature Portfolio wishes to improve the reproducibility of the work that we publish. This form provides structure for consistency and transparency in reporting. For further information on Nature Portfolio policies, see our Editorial Policies and the Editorial Policy Checklist.

Statistics

For all statistical analyses, confirm that the following items are present in the figure legend, table legend, main text, or Methods section.

n/a	Confirmed	
☐	☒	The exact sample size (*n*) for each experimental group/condition, given as a discrete number and unit of measurement
☐	☒	A statement on whether measurements were taken from distinct samples or whether the same sample was measured repeatedly
☐	☒	The statistical test(s) used AND whether they are one- or two-sided<br>*Only common tests should be described solely by name; describe more complex techniques in the Methods section.*
☒	☐	A description of all covariates tested
☐	☒	A description of any assumptions or corrections, such as tests of normality and adjustment for multiple comparisons
☐	☒	A full description of the statistical parameters including central tendency (e.g. means) or other basic estimates (e.g. regression coefficient) AND variation (e.g. standard deviation) or associated estimates of uncertainty (e.g. confidence intervals)
☐	☒	For null hypothesis testing, the test statistic (e.g. *F*, *t*, *r*) with confidence intervals, effect sizes, degrees of freedom and *P* value noted<br>*Give P values as exact values whenever suitable.*
☒	☐	For Bayesian analysis, information on the choice of priors and Markov chain Monte Carlo settings
☒	☐	For hierarchical and complex designs, identification of the appropriate level for tests and full reporting of outcomes
☐	☒	Estimates of effect sizes (e.g. Cohen's *d*, Pearson's *r*), indicating how they were calculated

Our web collection on statistics for biologists contains articles on many of the points above.

Software and code

Policy information about availability of computer code

Data collection	Roche LightCycler 480 System (Roche), CytoFLEX LX (Beckman Coulter), ZEISS LSM 880 confocal microscope equipped with fast airyscan (Carl Zeiss AG), ChemiDoc Touch Imaging System (Bio-Rad), Q ExactiveTM Mass Spectrometer (ThermoFisher Scientific). Multifunctional Microplate Reader (Tecan).
Data analysis	Image Lab version 4.1 (Bio-Rad) was used to acquire Immunoblots and protein Coomassie staining gels. Immunofluorescence images were analyzed using ZEISS ZEN 2.6 blue edition software (Carl Zeiss AG). Apoptosis data was analyzed using CytExpert software (Beckman Coulter). Statistical analysis was performed with GraphPad Prism version 8.3.0 (La Jolla, CA, USA) and IBM SPSS Statistics version 21.0 (Armonk, NY, USA). The MS data were analyzed using MASCOT(http://www.matrixscience.com/).

For manuscripts utilizing custom algorithms or software that are central to the research but not yet described in published literature, software must be made available to editors and reviewers. We strongly encourage code deposition in a community repository (e.g. GitHub). See the Nature Portfolio guidelines for submitting code & software for further information.

Data

Policy information about availability of data

All manuscripts must include a data availability statement. This statement should provide the following information, where applicable:

- Accession codes, unique identifiers, or web links for publicly available datasets
- A description of any restrictions on data availability
- For clinical datasets or third party data, please ensure that the statement adheres to our policy

Analysis of the expression of candidate genes from the CRISPR–Cas9 screen in colon and rectum adenocarcinoma patients was performed with GEPIA database (http://gepia2.cancer-pku.cn/#analysis). The putative NLS in GCLM was predicted by cNLS Mapper (https://nls-mapper.iab.keio.ac.jp/cgi-bin/NLS_Mapper_form.cgi). The putative phosphorylation sites in GCLM were analyzed using PhosphoSitePlus (https://www.phosphosite.org/proteinAction.action?id=4849775&showAllSites=true). The sequencing raw data related to the CRISPR–Cas9 screen results has been deposited to Gene Expression Omnibus under the accession number GSE254136 (https://www.ncbi.nlm.nih.gov/geo/query/acc.cgi?acc=GSE254136). The mass spectrometry raw data have been deposited to the ProteomeXchange Consortium via the PRIDE partner repository with the dataset identifier PXD055376 (http://www.ebi.ac.uk/pride/archive/projects/PXD055376). The antibodies and reagents used in this study are listed in Supplementary Data 4.

Research involving human participants, their data, or biological material

Policy information about studies with human participants or human data. See also policy information about sex, gender (identity/presentation), and sexual orientation and race, ethnicity and racism.

Reporting on sex and gender	The patients included males and females, and the information on sex and gender was not relevant in our study.
Reporting on race, ethnicity, or other socially relevant groupings	N/A
Population characteristics	We obtained 406 pairs of normal versus CRC tissue specimens (163 female, 243 male, average age: 56 ± 12 years), 58 CRC tissues with FOLFOX or XELOX chemotherapy (tissue samples are collected before treatment) (26 female, 32 male, average age: 54 ± 12 years), 235 GC tissue (79 female, 156 male, average age: 55 ± 12 years) and 276 ESCC tissue (78 female, 198 male, average age: 57 ± 9 years) for IHC staining analysis from SYSUCC.
Recruitment	All CRC、GC、ESCC patient samples were obtained from Sun Yat-sen University Cancer Center (SYSUCC, Guangzhou, China) after appropriate review of our research project to ensure that it was covered under their ethics approval (G2021-029-01). All patient samples were collected by SUSYCC based on availability and informed consent provided by the individuals. All patients were treated with standard of care according to treatment guidelines and were followed up on a regular basis. Baseline characteristic of samples were balanced among groups without bias.
Ethics oversight	The study protocol was approved by the Institutional Review Board of Sun Yat-sen University Cancer Center (G2021-029-01). Written informed consent was obtained from the patients who provided samples.

Note that full information on the approval of the study protocol must also be provided in the manuscript.

Field-specific reporting

Please select the one below that is the best fit for your research. If you are not sure, read the appropriate sections before making your selection.

[x] Life sciences [ ] Behavioural & social sciences [ ] Ecological, evolutionary & environmental sciences

For a reference copy of the document with all sections, see nature.com/documents/nr-reporting-summary-flat.pdf

Life sciences study design

All studies must disclose on these points even when the disclosure is negative.

Sample size	No statistical method was used to predetermine sample size. Sample size was chosen based on previous experience and standards in the field (Jeon S-M, Chandel NS, Hay N. Nature 485, 661-665 (2012). Liu X, et al. Nat Cell Biol 18, 431-442 (2016)). For example, all of the experiments were repeated at least 3 times, and the sample sizes for in vitro (n >= 3) and in vivo (n >= 5) .
Data exclusions	No data were excluded for analysis.
Replication	For each representative image/data, experiments were performed at least three times with similar results unless otherwise noted in the manuscript.
Randomization	All the samples, organisms and participants were randomly allocated into different experimental groups.
Blinding	Investigators were not blinded to treatments due to the unbiased nature of molecular biological assays used, but no subjective assessments were made.

Behavioural & social sciences study design

All studies must disclose on these points even when the disclosure is negative.

Study description	*Briefly describe the study type including whether data are quantitative, qualitative, or mixed-methods (e.g. qualitative cross-sectional, quantitative experimental, mixed-methods case study).*
Research sample	*State the research sample (e.g. Harvard university undergraduates, villagers in rural India) and provide relevant demographic information (e.g. age, sex) and indicate whether the sample is representative. Provide a rationale for the study sample chosen. For studies involving existing datasets, please describe the dataset and source.*
Sampling strategy	*Describe the sampling procedure (e.g. random, snowball, stratified, convenience). Describe the statistical methods that were used to predetermine sample size OR if no sample-size calculation was performed, describe how sample sizes were chosen and provide a rationale for why these sample sizes are sufficient. For qualitative data, please indicate whether data saturation was considered, and what criteria were used to decide that no further sampling was needed.*
Data collection	*Provide details about the data collection procedure, including the instruments or devices used to record the data (e.g. pen and paper, computer, eye tracker, video or audio equipment) whether anyone was present besides the participant(s) and the researcher, and whether the researcher was blind to experimental condition and/or the study hypothesis during data collection.*
Timing	*Indicate the start and stop dates of data collection. If there is a gap between collection periods, state the dates for each sample cohort.*
Data exclusions	*If no data were excluded from the analyses, state so OR if data were excluded, provide the exact number of exclusions and the rationale behind them, indicating whether exclusion criteria were pre-established.*
Non-participation	*State how many participants dropped out/declined participation and the reason(s) given OR provide response rate OR state that no participants dropped out/declined participation.*
Randomization	*If participants were not allocated into experimental groups, state so OR describe how participants were allocated to groups, and if allocation was not random, describe how covariates were controlled.*

Ecological, evolutionary & environmental sciences study design

All studies must disclose on these points even when the disclosure is negative.

Study description	*Briefly describe the study. For quantitative data include treatment factors and interactions, design structure (e.g. factorial, nested, hierarchical), nature and number of experimental units and replicates.*
Research sample	*Describe the research sample (e.g. a group of tagged Passer domesticus, all Stenocereus thurberi within Organ Pipe Cactus National Monument), and provide a rationale for the sample choice. When relevant, describe the organism taxa, source, sex, age range and any manipulations. State what population the sample is meant to represent when applicable. For studies involving existing datasets, describe the data and its source.*
Sampling strategy	*Note the sampling procedure. Describe the statistical methods that were used to predetermine sample size OR if no sample-size calculation was performed, describe how sample sizes were chosen and provide a rationale for why these sample sizes are sufficient.*
Data collection	*Describe the data collection procedure, including who recorded the data and how.*
Timing and spatial scale	*Indicate the start and stop dates of data collection, noting the frequency and periodicity of sampling and providing a rationale for these choices. If there is a gap between collection periods, state the dates for each sample cohort. Specify the spatial scale from which the data are taken*
Data exclusions	*If no data were excluded from the analyses, state so OR if data were excluded, describe the exclusions and the rationale behind them, indicating whether exclusion criteria were pre-established.*
Reproducibility	*Describe the measures taken to verify the reproducibility of experimental findings. For each experiment, note whether any attempts to repeat the experiment failed OR state that all attempts to repeat the experiment were successful.*
Randomization	*Describe how samples/organisms/participants were allocated into groups. If allocation was not random, describe how covariates were controlled. If this is not relevant to your study, explain why.*
Blinding	*Describe the extent of blinding used during data acquisition and analysis. If blinding was not possible, describe why OR explain why blinding was not relevant to your study.*

Did the study involve field work? ☐ Yes ☐ No

Field work, collection and transport

Field conditions	*Describe the study conditions for field work, providing relevant parameters (e.g. temperature, rainfall).*
Location	*State the location of the sampling or experiment, providing relevant parameters (e.g. latitude and longitude, elevation, water depth).*
Access & import/export	*Describe the efforts you have made to access habitats and to collect and import/export your samples in a responsible manner and in compliance with local, national and international laws, noting any permits that were obtained (give the name of the issuing authority, the date of issue, and any identifying information).*
Disturbance	*Describe any disturbance caused by the study and how it was minimized.*

Reporting for specific materials, systems and methods

We require information from authors about some types of materials, experimental systems and methods used in many studies. Here, indicate whether each material, system or method listed is relevant to your study. If you are not sure if a list item applies to your research, read the appropriate section before selecting a response.

Materials & experimental systems

n/a	Involved in the study	
☐	☒	Antibodies
☐	☒	Eukaryotic cell lines
☒	☐	Palaeontology and archaeology
☐	☒	Animals and other organisms
☒	☐	Clinical data
☒	☐	Dual use research of concern
☒	☐	Plants

Methods

n/a	Involved in the study	
☒	☐	ChIP-seq
☒	☐	Flow cytometry
☒	☐	MRI-based neuroimaging

Antibodies

Antibodies used

Rabbit monoclonal anti-GCLM, Abcam, Cat# ab126704,
Rabbit polyclonal anti-GCLM,Abcam, Cat# ab154017,
Rabbit polyclonal anti-NKRF, Abcam, Cat# ab83977,
Rabbit polyclonal anti-NKRF, Abcam, Cat# ab168829,
Rabbit monoclonal anti-Lamin B1, Abcam , Cat# ab133741,
Rabbit monoclonal anti-Phosphothreonine, Abcam, Cat# ab218195,
Rabbit polyclonal anti-Phosphoserine, Abcam, Cat# ab9332,
Mouse monoclonal anti-Phosphotyrosine, Abcam, Cat# ab10321,
Rabbit monoclonal anti- NF-κB p65, Abcam, Cat# ab32536,
Rabbit monoclonal anti- NF-κB p105/p50, Abcam, Cat# ab32360,
Rabbit polyclonal anti-His tag, Abcam, Cat# ab9108,
Rabbit monoclonal anti-ATM phospho S1981, Abcam, Cat# ab81292,
Rabbit monoclonal anti-NF-κB, Cell Signaling Technology, Cat# 8242,
Rabbit monoclonal anti-α-Tubulin, Cell Signaling Technology, Cat# 2125,
Mouse monoclonal anti-GST-Tag, Cell Signaling Technology, Cat# 2624,
Rabbit polyclonal anti-Ubiquitin, Cell Signaling Technology, Cat# 3933,
Goat Anti-rabbit IgG (H+L), F(ab')2 Fragment (Alexa Fluor® 488 Conjugate) , Cell Signaling Technology, Cat# 4412 ,
Rabbit polyclonal anti-Phospho-p38 MAPK Thr180/Tyr182, Cell Signaling Technology, Cat# 9211,
Goat anti-Mouse IgG (H+L) Cross-Adsorbed ReadyProbes Secondary Antibody, Alexa Fluor™ 594, Thermo Fisher Scientific, Cat# R37121
Rabbit polyclonal anti-importin α5, Proteintech, Cat# 18137-1-AP,
Mouse monoclonal anti-importin α5 , Proteintech, Cat# 67897-1-Ig,
Mouse monoclonal anti-Ki67, ZSGB-Bio, Cat# ZM-0167,
Rabbit polyclonal anti-Pan-acetyllysine, PTM BIO, Cat# PTM-105,
Rabbit polyclonal anti-Mono/Di-Methyllysine, PTM BIO, Cat# PTM-602,
Normal mouse IgG, Santa Cruz Biotechnology, Cat# sc-2025,
Mouse monoclonal anit-Flag-Tag, Dia-An Biotechnology, Cat# 2064,
Mouse monoclonal anti-HA-Tag, Dia-An Biotechnology, Cat# 2063,
Rabbit polyclonal anti-Phospho-T17-GCLM, Dia-An Biotechnology, N/A
Rabbit polyclonal anti-Phospho-T106-GCLM, Dia-An Biotechnology, N/A
Rabbit polyclonal anti-Phospho-T164-GCLM, Dia-An Biotechnology, N/A

Validation	Rabbit monoclonal anti-GCLM, Abcam, Cat# ab126704, Supplier validation: "Applications: Western Blot, Immunohistochemistry; Reactivity Notes: Mouse, Rat, Human”.<br>Rabbit polyclonal anti-GCLM,Abcam, Cat# ab154017, Supplier validation: "Applications: WB, IHC-P, ICC/IF; Reactivity Notes: Human”.<br>Rabbit polyclonal anti-NKRF, Abcam, Cat# ab83977, Supplier validation: "Applications: Western Blot, Immunohistochemistry; Reactivity Notes: Human”.<br>Rabbit polyclonal anti-NKRF, Abcam, Cat# ab168829, Supplier validation: "Applications: IHC-P, WB, IP; Reactivity Notes: Human”.<br>Rabbit monoclonal anti-Lamin B1, Abcam , Cat# ab133741, Supplier validation: "Applications: Western Blot, Immunohistochemistry, Immunoprecipitation, Immunofluorescence; Reactivity Notes: Mouse, Rat, Human”.<br>Rabbit monoclonal anti-Phosphothreonine, Abcam, Cat# ab218195, Supplier validation: "Applications: Western Blot, ELISA; Reactivity Notes: Mouse, Rat, Human, Recombinant fragment”.<br>Rabbit polyclonal anti-Phosphoserine, Abcam, Cat# ab9332, Supplier validation: "Applications: Western Blot; Reactivity Notes: Species independent.<br>Mouse monoclonal anti-Phosphotyrosine, Abcam, Cat# ab10321, Supplier validation: "Applications: Western Blot, Immunofluorescence; Reactivity Notes: Species independent”.<br>Rabbit monoclonal anti- NF-κB p65, Abcam, Cat# abab32536, Supplier validation: "Applications: WB, IHC-P, ICC/IF, IP; Reactivity Notes: Mouse, Human”.<br>Rabbit monoclonal anti- NF-κB p105/p50, Abcam, Cat# ab32360, Supplier validation: "Applications: Western Blot, Immunohistochemistry; Reactivity Notes: Mouse, Rat, Human”.<br>Rabbit polyclonal anti-His tag, Abcam, Cat# ab9108, Supplier validation: "Applications: Western Blot, Immunofluorescence, ELISA, Chromatin Immunoprecipitation; Reactivity Notes: Species independent”.<br>Rabbit monoclonal anti-ATM phospho S1981, Abcam, Cat# ab81292, Supplier validation: "Applications: Dot Blot, Flow Cytometry, Western Blot, Immunohistochemistry; Reactivity Notes: Human”.<br>Mouse monoclonal Anti-β-Actin ,Sigma-Aldrich, Cat# A5441, Supplier validation: "Applications: Western Blot, Immunohistochemistry, Immunofluorescence; Reactivity Notes: Sheep, Carp, Feline, Chicken, Rat, Mouse, Hirudo medicinalis, Rabbit, Canine, Pig, Human, Bovine, Guinea pig”.<br>Rabbit monoclonal anti-NF-κB, Cell Signaling Technology, Cat# 8242, Supplier validation: "Applications: Western Blot, Immunoprecipitation, Immunofluorescence, Flow Cytometry, Chromatin Immunoprecipitation, Chromatin Immunoprecipitation Sequencing, CUT&RUN, Simple Western, Immunohistochemistry; Reactivity Notes: Human, Mouse, Rat, Hamster dog”.<br>Rabbit monoclonal anti-α-Tubulin, Cell Signaling Technology, Cat# 2125, Supplier validation: "Applications: Western Blot, Simple Western, Immunohistochemistry, Immunofluorescence, Flow Cytometry; Reactivity Notes: Human, Mouse, Rat, Monkey D.melanogaster, Zebrafish, Bovine, Pig”.<br>Mouse monoclonal anti-GST-Tag, Cell Signaling Technology, Cat# 2624, Supplier validation: "Applications: Western Blot, Immunoprecipitation, Immunofluorescence; Reactivity Notes: All Species Expected”.<br>Rabbit polyclonal anti-Ubiquitin, Cell Signaling Technology, Cat# 3933, Supplier validation: "Applications: Western Blot, Immunohistochemistry; Reactivity Notes: All Species Expected”.<br>Goat Anti-rabbit IgG (H+L), F(ab')2 Fragment (Alexa Fluor® 488 Conjugate) , Cell Signaling Technology, Cat# 4412, Supplier validation: "Applications: IF-IC, F; Reactivity Notes:Rabbit”.<br>Goat anti-Mouse IgG (H+L) Cross-Adsorbed ReadyProbes Secondary Antibody, Alexa Fluor™ 594, Thermo Fisher Scientific, Cat# R37121, Supplier validation: "Applications: ICC/IF, Flow; Reactivity Notes:Mouse”.<br>Rabbit polyclonal anti-Phospho-p38 MAPK Thr180/Tyr182, Cell Signaling Technology, Cat# 9211, Supplier validation: "Applications: Western Blot, Immunohistochemistry, Immunofluorescence; Reactivity Notes: Human, Mouse, Rat, Monkey, D.melanogaster, Pig, S.cerevisiae”.<br>Rabbit polyclonal anti-importin α5, Proteintech, Cat# 18137-1-AP, Supplier validation: "Applications: Western Blot, Immunohistochemistry, Immunofluorescence, Immunoprecipitation, ELISA; Reactivity Notes: Mouse, Rat, Human”.<br>Mouse monoclonal anti-importin α5 , Proteintech, Cat# 67897-1-Ig, Supplier validation: "Applications: WB, IHC, IF/ICC, ELISA; Reactivity Notes: Human, mouse, rat samples”.<br>Mouse monoclonal anti-Ki67, ZSGB-Bio, Cat# ZM-0167, Supplier validation: "Applications: Immunohistochemistry; Reactivity Notes: Human”.<br>Rabbit polyclonal anti-Pan-acetyllysine, PTM BIO, Cat# PTM-105, Supplier validation: "Applications: Western Blot, Immunohistochemistry, Immunofluorescence, Immunoprecipitation, Chromatin Immunoprecipitation; Reactivity Notes: All”.<br>Rabbit polyclonal anti-Mono/Di-Methyllysine, PTM BIO, Cat# PTM-602, Supplier validation: "Applications: Western Blot; Reactivity Notes: All”.<br>Normal mouse IgG, Santa Cruz Biotechnology, Cat# sc-2025, Supplier validation: "Applications: Western Blot, Immunohistochemistry, Immunoprecipitation”.<br>Mouse monoclonal anit-Flag-Tag, Dia-An Biotechnology, Cat# 2064, Supplier validation: "Applications: Western Blot, Immunohistochemistry, ELISA, Immunoprecipitation, Immunofluorescence, Chromatin Immunoprecipitation; Reactivity Notes: Human, Mouse, Rat, Zebra fish”.<br>Mouse monoclonal anti-HA-Tag, Dia-An Biotechnology, Cat# 2063, Supplier validation: "Applications: Western Blot, Immunoprecipitation, Immunofluorescence; Reactivity Notes: Protein fused with HA tag”.

Eukaryotic cell lines

Policy information about cell lines and Sex and Gender in Research

Cell line source(s)	The human embryonic kidney (HEK) 293T (Cat# CRL-11268), human HCT116 (Cat# CRL-247), and DLD1 (Cat# CRL-221) CRC cell lines were purchased from the American Type Culture Collection (ATCC, Manassas, VA, USA).
Authentication	All cell lines were authenticated based on STR fingerprinting before use.
Mycoplasma contamination	All cell lines were tested negative for mycoplasma contamination. Stated in the Methods section.

Commonly misidentified lines (See ICLAC register)	No commonly misidentified cell lines were used.

Palaeontology and Archaeology

Specimen provenance	*Provide provenance information for specimens and describe permits that were obtained for the work (including the name of the issuing authority, the date of issue, and any identifying information). Permits should encompass collection and, where applicable, export.*
Specimen deposition	*Indicate where the specimens have been deposited to permit free access by other researchers.*
Dating methods	*If new dates are provided, describe how they were obtained (e.g. collection, storage, sample pretreatment and measurement), where they were obtained (i.e. lab name), the calibration program and the protocol for quality assurance OR state that no new dates are provided.*

☐ Tick this box to confirm that the raw and calibrated dates are available in the paper or in Supplementary Information.

Ethics oversight	*Identify the organization(s) that approved or provided guidance on the study protocol, OR state that no ethical approval or guidance was required and explain why not.*

Note that full information on the approval of the study protocol must also be provided in the manuscript.

Animals and other research organisms

Policy information about studies involving animals; ARRIVE guidelines recommended for reporting animal research, and Sex and Gender in Research

Laboratory animals	The BALB/c nude mice (6 to 8 weeks old,half male and half female) used in these experiments were obtained from Beijing Vital River Laboratory (Beijing, China) and the six-week-old mice with intestine-specific Gclm knockout (GclmiKO) in the C57BL/6J background were purchased from Gempharmatech (Jiangsu, China). Mice were housed in temperature-controlled pathogen-free conditions (around 20℃, 40% humidity) under a 12-h light/dark cycle. All animal experiments were performed based on the protocol approved by Institutional Ethics Committee for Clinical Research and Animal Trials of the SYSUCC (L102012021001X).
Wild animals	The study did not involve wild animals
Reporting on sex	Sex is not relevant in this study. Our preliminary experiments showed that GCLM knockdown phenotypes were identical in male and female mice and only female mice were used for the following experiments to control variables.
Field-collected samples	This study did not include field-collected samples.
Ethics oversight	All animal studies were performed in accordance with a protocol approved by the Institutional Ethics Committee for Clinical Research and Animal Trials of the Sun Yat-sen University Cancer Center (L102012021001X).

Note that full information on the approval of the study protocol must also be provided in the manuscript.

Clinical data

Policy information about clinical studies

All manuscripts should comply with the ICMJEguidelines for publication of clinical research and a completedCONSORT checklist must be included with all submissions.

Clinical trial registration	*Provide the trial registration number from ClinicalTrials.gov or an equivalent agency.*
Study protocol	*Note where the full trial protocol can be accessed OR if not available, explain why.*
Data collection	*Describe the settings and locales of data collection, noting the time periods of recruitment and data collection.*
Outcomes	*Describe how you pre-defined primary and secondary outcome measures and how you assessed these measures.*

Dual use research of concern

Policy information about dual use research of concern

Hazards

Could the accidental, deliberate or reckless misuse of agents or technologies generated in the work, or the application of information presented in the manuscript, pose a threat to:

No	Yes	
☐	☐	Public health
☐	☐	National security
☐	☐	Crops and/or livestock
☐	☐	Ecosystems
☐	☐	Any other significant area

Experiments of concern

Does the work involve any of these experiments of concern:

No	Yes	
☐	☐	Demonstrate how to render a vaccine ineffective
☐	☐	Confer resistance to therapeutically useful antibiotics or antiviral agents
☐	☐	Enhance the virulence of a pathogen or render a nonpathogen virulent
☐	☐	Increase transmissibility of a pathogen
☐	☐	Alter the host range of a pathogen
☐	☐	Enable evasion of diagnostic/detection modalities
☐	☐	Enable the weaponization of a biological agent or toxin
☐	☐	Any other potentially harmful combination of experiments and agents

Plants

Seed stocks	*Report on the source of all seed stocks or other plant material used. If applicable, state the seed stock centre and catalogue number. If plant specimens were collected from the field, describe the collection location, date and sampling procedures.*
Novel plant genotypes	*Describe the methods by which all novel plant genotypes were produced. This includes those generated by transgenic approaches, gene editing, chemical/radiation-based mutagenesis and hybridization. For transgenic lines, describe the transformation method, the number of independent lines analyzed and the generation upon which experiments were performed. For gene-edited lines, describe the editor used, the endogenous sequence targeted for editing, the targeting guide RNA sequence (if applicable) and how the editor was applied.*
Authentication	*Describe any authentication procedures for each seed stock used or novel genotype generated. Describe any experiments used to assess the effect of a mutation and, where applicable, how potential secondary effects (e.g. second site T-DNA insertions, mosiacism, off-target gene editing) were examined.*

ChIP-seq

Data deposition

☐ Confirm that both raw and final processed data have been deposited in a public database such as GEO.

☐ Confirm that you have deposited or provided access to graph files (e.g. BED files) for the called peaks.

Data access links<br>*May remain private before publication.*	*For "Initial submission" or "Revised version" documents, provide reviewer access links. For your "Final submission" document, provide a link to the deposited data.*
Files in database submission	*Provide a list of all files available in the database submission.*
Genome browser session<br>(e.g. UCSC)	*Provide a link to an anonymized genome browser session for "Initial submission" and "Revised version" documents only, to enable peer review. Write "no longer applicable" for "Final submission" documents.*

Methodology

Replicates	*Describe the experimental replicates, specifying number, type and replicate agreement.*
Sequencing depth	*Describe the sequencing depth for each experiment, providing the total number of reads, uniquely mapped reads, length of reads and whether they were paired- or single-end.*
Antibodies	*Describe the antibodies used for the ChIP-seq experiments; as applicable, provide supplier name, catalog number, clone name, and lot number.*
Peak calling parameters	*Specify the command line program and parameters used for read mapping and peak calling, including the ChIP, control and index files used.*

Data quality

Describe the methods used to ensure data quality in full detail, including how many peaks are at FDR 5% and above 5-fold enrichment.

Software

Describe the software used to collect and analyze the ChIP-seq data. For custom code that has been deposited into a community repository, provide accession details.

Flow Cytometry

Plots

Confirm that:

☐ The axis labels state the marker and fluorochrome used (e.g. CD4-FITC).

☐ The axis scales are clearly visible. Include numbers along axes only for bottom left plot of group (a 'group' is an analysis of identical markers).

☐ All plots are contour plots with outliers or pseudocolor plots.

☐ A numerical value for number of cells or percentage (with statistics) is provided.

Methodology

Sample preparation

Describe the sample preparation, detailing the biological source of the cells and any tissue processing steps used.

Instrument

Software

Cell population abundance

Describe the abundance of the relevant cell populations within post-sort fractions, providing details on the purity of the samples and how it was determined.

Gating strategy

Describe the gating strategy used for all relevant experiments, specifying the preliminary FSC/SSC gates of the starting cell population, indicating where boundaries between "positive" and "negative" staining cell populations are defined.

☐ Tick this box to confirm that a figure exemplifying the gating strategy is provided in the Supplementary Information.

Magnetic resonance imaging

Experimental design

Design type

Indicate task or resting state; event-related or block design.

Design specifications

Specify the number of blocks, trials or experimental units per session and/or subject, and specify the length of each trial or block (if trials are blocked) and interval between trials.

Behavioral performance measures

State number and/or type of variables recorded (e.g. correct button press, response time) and what statistics were used to establish that the subjects were performing the task as expected (e.g. mean, range, and/or standard deviation across subjects).

Acquisition

Imaging type(s)

Specify: functional, structural, diffusion, perfusion.

Field strength

Specify in Tesla

Sequence & imaging parameters

Specify the pulse sequence type (gradient echo, spin echo, etc.), imaging type (EPI, spiral, etc.), field of view, matrix size, slice thickness, orientation and TE/TR/flip angle.

Area of acquisition

State whether a whole brain scan was used OR define the area of acquisition, describing how the region was determined.

Diffusion MRI ☐ Used ☐ Not used

Preprocessing

Preprocessing software

Provide detail on software version and revision number and on specific parameters (model/functions, brain extraction, segmentation, smoothing kernel size, etc.).

Normalization

If data were normalized/standardized, describe the approach(es): specify linear or non-linear and define image types used for transformation OR indicate that data were not normalized and explain rationale for lack of normalization.

Normalization template	*Describe the template used for normalization/transformation, specifying subject space or group standardized space (e.g. original Talairach, MNI305, ICBM152) OR indicate that the data were not normalized.*
Noise and artifact removal	*Describe your procedure(s) for artifact and structured noise removal, specifying motion parameters, tissue signals and physiological signals (heart rate, respiration).*
Volume censoring	*Define your software and/or method and criteria for volume censoring, and state the extent of such censoring.*

Statistical modeling & inference

Model type and settings	*Specify type (mass univariate, multivariate, RSA, predictive, etc.) and describe essential details of the model at the first and second levels (e.g. fixed, random or mixed effects; drift or auto-correlation).*
Effect(s) tested	*Define precise effect in terms of the task or stimulus conditions instead of psychological concepts and indicate whether ANOVA or factorial designs were used.*

Specify type of analysis: ☐ Whole brain ☐ ROI-based ☐ Both

Statistic type for inference<br>(See Eklund et al. 2016)	*Specify voxel-wise or cluster-wise and report all relevant parameters for cluster-wise methods.*
Correction	*Describe the type of correction and how it is obtained for multiple comparisons (e.g. FWE, FDR, permutation or Monte Carlo).*

Models & analysis

n/a	Involved in the study
☐	☐ Functional and/or effective connectivity
☐	☐ Graph analysis
☐	☐ Multivariate modeling or predictive analysis

Functional and/or effective connectivity	*Report the measures of dependence used and the model details (e.g. Pearson correlation, partial correlation, mutual information).*
Graph analysis	*Report the dependent variable and connectivity measure, specifying weighted graph or binarized graph, subject- or group-level, and the global and/or node summaries used (e.g. clustering coefficient, efficiency, etc.).*
Multivariate modeling and predictive analysis	*Specify independent variables, features extraction and dimension reduction, model, training and evaluation metrics.*
