# Supplementary material for: Nucleus-translocated GCLM promotes chemoresistance in colorectal cancer through a moonlighting function
Source: Nat Commun. 2025 Jan 2;16:263. doi: 10.1038/s41467-024-55568-1 (PMC11696352; doi:10.1038/s41467-024-55568-1)
Supplement: Supplementary file 7 — Description of Additional Supplementary Files [file 41467_2024_55568_MOESM7_ESM.pdf]

Supplementary Data 1:
List of CRISPR screen data.

Supplementary Data 2:
List of proteins bound by nuclear GCLM via LC-MS-MS analysis.

Supplementary Data 3:
List of proteins bound by total GCLM via LC-MSMS analysis.

Supplementary Data 4:
Sequence of siRNA, shRNA and sgRNA, primers and antibodies, reagent, kits and cells used in this study.
